# Cannabinoid 1/2 Receptor Activation Induces Strain-Dependent Behavioral and Neurochemical Changes in Genetic Absence Epilepsy Rats From Strasbourg and Non-epileptic Control Rats

**DOI:** 10.3389/fncel.2022.886033

**Published:** 2022-05-23

**Authors:** Philippe De Deurwaerdère, Maurizio Casarrubea, Daniel Cassar, Manuela Radic, Emilie Puginier, Abdeslam Chagraoui, Giuseppe Crescimanno, Vincenzo Crunelli, Giuseppe Di Giovanni

**Affiliations:** ^1^Centre National de la Recherche Scientifique, UMR 5287, Bordeaux Cedex, France; ^2^Laboratory of Behavioral Physiology, Department of Biomedicine, Neuroscience and Advanced Diagnostics (Bi.N.D.), Human Physiology Section “Giuseppe Pagano”, University of Palermo, Palermo, Italy; ^3^Laboratory of Neurophysiology, Department of Physiology and Biochemistry, Faculty of Medicine and Surgery, University of Malta, Msida, Malta; ^4^Department of Biomedical Sciences, Section of Neuroscience and Clinical Pharmacology, University of Cagliari, Cagliari, Italy; ^5^Normandie Université, UNIROUEN, INSERM, U1239, CHU Rouen, Neuronal and Neuroendocrine Differentiation and Communication Laboratory, Institute for Research and Innovation in Biomedicine of Normandy (IRIB), Rouen, France; ^6^Department of Medical Biochemistry, Rouen University Hospital, Rouen, France; ^7^Neuroscience Division, School of Biosciences, Cardiff University, Cardiff, United Kingdom

**Keywords:** cannabinoid receptors, CB1, CB2, strain-dependent effects, high-pressure liquid chromatography, thalamus, GABA, glutamate

## Abstract

Childhood absence epilepsy (CAE) is characterized by absence seizures, which are episodes of lack of consciousness accompanied by electrographic spike-wave discharges. About 60% of children and adolescents with absence seizures are affected by major neuropsychological comorbidities, including anxiety. Endocannabinoids and monoamines are likely involved in the pathophysiology of these CAE psychiatric comorbidities. Here, we show that the synthetic cannabinoid receptor type 1/2 (CB1/2R) agonist WIN 55,212-2 (2 mg/kg) has a strain-dependent effect on anxiety-like and motor behavior when assess in the hole board test and cerebral monoaminergic levels in Genetic Absence Epilepsy Rats from Strasbourg (GAERS) and their non-epileptic control (NEC) rat strain. Using quantitative and Temporal pattern (T-pattern) analyses, we found that WIN 55,212-2 did not affect the emotional status of GAERS, but it was anxiolytic in NEC. Conversely, WIN 55,212-2 had a sedative effect in GAERS but was ineffective in NEC. Moreover, vehicle-treated GAERS more motivated to explore by implementing more complex and articulated strategies. These behavioral changes correlate with the reduction of 5-HT in the hippocampus and substantia nigra (SN) and noradrenaline (NA) in the entopeduncular nucleus (EPN) in vehicle-treated GAERS compared to NEC rats, which could contribute to their low anxiety status and hypermotility, respectively. On the other hand, the increased level of NA in the EPN and 5-HT in the SN is consistent with an activation of the basal ganglia output-mediated motor suppression observed in WIN 55,212-2-treated GAERS rats. These data support the view of a strain-dependent alteration of the endocannabinoid system in absence epilepsy by adding evidence of a lower emotional responsiveness and a basal ganglia hypersensitivity to cannabinoids in GAERS compared to NEC rats.

## Introduction

Nearly 30% of people with convulsive epilepsy suffer from comorbid neuropsychiatric disorders, with the predominance of anxiety and depression (Mula et al., 2021). The presence of psychiatric comorbidities is a negative prognostic marker that is associated with a worse response to treatment, increased morbidity, mortality, and adverse clinical outcome (Michaelis et al., 2021). The burden of the commodities is even higher in people with non-convulsive epilepsy. Indeed, recent studies have demonstrated that the majority (60%) of children with childhood absence epilepsy (CAE) also suffer from various neuropsychiatric comorbidities (Glauser et al., 2013; Cnaan et al., 2017), including anxiety, mood disorders, and attention and learning deficits, which may precede the epilepsy diagnosis and persist, or even be aggravated, after full pharmacological control of the seizures (Crunelli et al., 2020; Gruenbaum et al., 2021).

Therefore, a major goal of translational research for children and teenagers with absence seizures (ASs) is to identify new treatments for their psychiatric comorbidities that do not exacerbate the seizure burden, thus, improving patients’ overall health and wellbeing. To achieve this goal, we need to further our understanding of the bidirectional link between anxiety and epilepsy (Kanner, 2013) and the underlying mechanisms, genetic predisposition, neuronal excitability alterations, and anatomical pathways (Hingray et al., 2019) that these psychiatric and neurological disorders may share. For instance, ASs and anxiety are linked to different alterations in neurotransmitters, such as monoamines (Marescaux et al., 1992; Svob Strac et al., 2016; Venzi et al., 2016; Crunelli et al., 2020) and endocannabinoids (eCBs) (van Rijn et al., 2010; Perescis et al., 2020; Roebuck et al., 2020, 2021), which are likely to interact with each other (Colangeli et al., 2021). However, these interactions have not been studied yet in comorbid anxiety of animal models of CAE.

To shed some light on CAE anxiety, we used the Genetic Absence Epilepsy Rats from Strasburg (GAERS), a very well-validated polygenic model of absence epilepsy presenting both recurrent generalized non-convulsive seizures and neuropsychiatric comorbidities (Depaulis et al., 2016; Crunelli et al., 2020; Gruenbaum et al., 2021) and their non-epileptic control (NEC) rats (Vergnes et al., 1982; Marescaux et al., 1992; Depaulis et al., 2016) in the hole-board apparatus (Casarrubea et al., 2009a,b). Moreover, to study the possible interaction between the endocannabinoid system (ECS) and monoamines, we measured tissue concentration of dopamine (DA), serotonin (5-HT), noradrenaline (NA), and some of their metabolites (De Deurwaerdère et al., 2020; Di Giovanni et al., 2020) by HPLC in 14 brain structures of different age-matched groups of GAERS and NEC rats after acute systemic administration of the cannabinoid 1/2 receptor agonist (CB1/2R) WIN 55,212-2 (Howlett et al., 2002) and its vehicle.

To untangle CB1R-induced hypomotility (Zimmer et al., 1999) from any anxiety-like behavior effect, we used Temporal -pattern (T-pattern) detection and their multivariate analysis that has been shown to reveal hidden behavioral components and distinguish between different anxiety levels (Casarrubea et al., 2018, 2021b; Aiello et al., 2020). Unexpectedly, in vehicle-treated GAERS, a lower anxious state and reduced immobility were observed than in vehicle-treated NEC rats. The reduction of 5-HT in the dorsal hippocampus (dHP), NA in the entopeduncular nucleus (EPN), and 5-HT in the substantia nigra (SN) in vehicle-treated GAERS rats may explain their lower levels of anxiety and higher mobility, respectively, compared to NEC. WIN 55,212-2 had a mainly sedative effect but did not modify the emotionality of GAERS as revealed by T-pattern analysis. On the other hand, WIN 55,212-2 was anxiolytic in NEC, but it did not affect their motor behavior. These behavioral changes were paralleled by an increase of NA in the EPN and 5-HT in the SN, consistent with an activation of the inhibitory basal ganglia output and induced sedation in WIN 55,212-2-treated GAERS.

## Materials and Methods

**FIGURE 1:**
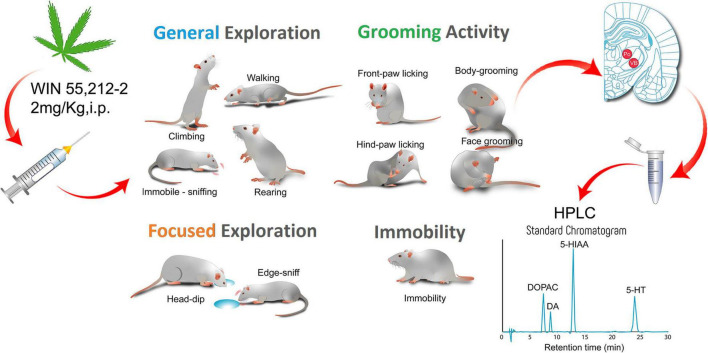
Graphical abstract summarizing the main aspects of the study. Adult male GAERS and NEC received an IP administration of 2 mg/kg of the CB1/2 receptor agonist WIN 55,212-2 and 30 min after were placed into the arena of the hole-board test to study the effect of the cannabinoid receptor activation on the neophilia and emotional behavior of the two inbred strains. We measured General Exploration, Focused Exploration, Grooming activity, and Immobility. We analyzed the data by quantitative and T-Pattern analysis. Different groups of animals received the same pharmacological treatment and after the 30 min from the injection their brains were removed and frozen for HPLC analysis of the monoamines dopamine (DA), noradrenaline and serotonin (5-HT) and some of their metabolites (3,4-Dihydroxyphenylacetic acid, DOPAC; 5-hydroxyindoleacetic acid, 5-HIAA). WIN 55,212-2 reduced motor behavior but did not affect emotionality of the epileptic GAERS rats and produced neurochemical changes in the hippocampus and basal ganglia.

The experimental approach of the *in vivo* and *ex vivo* study is depicted in Figure 1.

### Animals

Male GAERS and NEC rats (3–5 months old) were obtained from a colony bred at the University of Malta. Animals were housed in a 12:12 light cycle (lights on at 7:00 a.m. and off at 7:00 p.m.). All animal procedures were approved and carried out following University of Malta ethical guidelines and in conformity with Maltese and international laws and policies (EU Directive, 2010/63/EU for animal experiments, ARRIVE guidelines, and the Basel declaration including the 3R concept). All efforts were made to minimize animal suffering and to reduce the number of animals used.

### Pharmacological Treatment and Experimental Design

The (R)-(+)-[2,3-Dihydro-5-methyl-3-(4-morpholinylmethyl) pyrrolo[1,2,3-de]-1,4-benzoxazin-6-yl]-1-naphthalenylmetha- none mesylate, (R)-(+)-WIN 55,212-2, was purchased from Tocris Cookson Ltd. (Bristol, United Kingdom). WIN 55,212-2 was freshly dissolved in a vehicle solution (2 ml/kg) made of 5% PEG-400, 5% Tween 80 in saline, and was interpersonally (IP) administered.

### Behavioral Analyses

#### Experimental Apparatus

The hole-board apparatus used in the present study consisted of a square (50 × 50 cm) arena made of white opaque Plexiglas with a raised floor, containing four equidistant holes 4 cm in diameter. Each hole’s center has a 10-cm distance from the two adjacent Plexiglas walls and all the four holes were equidistant. The floor of the apparatus was positioned 5 cm above a white opaque Plexiglas sub-floor. The square arena was surrounded by three white opaque Plexiglas walls (50 × 50 cm) and a front transparent wall (50 × 50 cm). A digital video camera (model: Toshiba HDDV P10) was placed in front of the transparent wall to record the behavior of each rat. The behavior of each subject was recorded on the cam’s SD card. At the end of each day’s recording sessions, video files from the SD card were transferred and stored on a personal computer for the following analyses.

#### Experimental Procedure

On the day of the experiment, rats were allowed to acclimatize to the testing room for 30 min. Before the start of a new test or the use of a new subject, the apparatus was carefully cleaned with ethyl alcohol to remove possible scent cues left by the preceding animal. Thirty mins before the beginning of the test, WIN 55,212-2 (2 mg/kg) or its vehicle were IP-injected. Each subject, naïve to the test, was placed in the central area of the apparatus, was allowed to explore for 10 min and its behavior was recorded using a digital camera.

#### Data Analysis

A list of all the components of the behavioral repertoire and their formal description is shown in Supplementary Figure 1. The ethogram is based on the different components of various behavioral categories as analyzed in our previous studies (Casarrubea et al., 2009a,b; 2017, 2020), and it particularly encompasses eleven behavioral components divided into four main categories (Supplementary Figure 1). *General Exploration* encompasses all the activities of environmental exploration, excluding the holes: Walking (Wa), Climbing (Cl), Immobile Sniffing (IS), and Rearing (Re). *Focused Exploration* does contain the components aimed at the exploration of the ground holes: Edge-Sniff (ES), and Head-Dip (HD). *Grooming Activity* concerns Head-, Paws-, Body-, and Tail-related self-cleaning behaviors as follows: Front-Paw Licking (FPL), Hind-Paw Licking (HPL), Face Grooming (FG), and Body Grooming (BG). The last category contains only *Immobility* (Im). Based on this ethogram, video files of each rat were annotated by a trained observer that was blind to treatment and strain using a professional software tool (The Observer, Noldus Information Technology, Netherlands) and event log files were generated for each subject. The propaedeutic assessment of reliability was evaluated based on five video files, randomly taken from the experimental recordings, and scored by the observer in two different moments. In a second step, after such a propaedeutic step, all video files were annotated. Once the event log files of all subjects were obtained, they were re-arranged to contain exclusively performed behaviors and their onset. These files were then utilized to perform both quantitative and T-pattern analyses. The T-pattern analysis is a multivariate approach useful to reveal the temporal structure of behavior. Such a technique orbits around the detection of statistically significant time distances among events in time. The procedure encompasses a detection step followed by an analytical part and does require the utilization of a specific software tool known as Theme (PatternVision Ltd., Reykjavik, Iceland). In brief, given the distribution of events occurring within a hypothetical observation period ranging from Time 0 (T0) to time x (Tx) (i.e., T0-Tx), an algorithm compares the distributions of each pair of behavioral events, e.g., “a” and “b,” searching for a time window, so that “a” is followed by “b” within such a time window. If this condition between the event “a” and the event “b” exists and is verified, a first-level T-pattern including only two events that are (a b) detected, then, such a first level T-pattern is considered as a potential “a” or “b” terms for the detection of higher-order patterns, e.g., [(a b) c], and so on up to any level. When no more patterns are detected, the search stops. T-patterns can be indicated using textual strings, where behavioral events in brackets represent the textual representations of T-patterns in terms of order of events and brackets indicate hierarchical detections of events within each T-patterns based on the above-mentioned bottom-up detection process. Another approach to represent T-patterns, more intuitive but often requiring an excessive amount of space, is using tree representations, i.e., tree structures, similar to dendrograms, where connections among events are organized based on the hierarchical detections. Concepts, theories, and procedures concerning the detection and analysis of T-patterns have been extensively described previously (Casarrubea et al., 2015, 2018; Magnusson, 2020). The search parameters used are as follows: significance level = 0.0001; lumping factor = 0.90; and minimum percent of samples = 100%. For each group, the following behavioral responses were analyzed: mean occurrences and mean duration of each behavioral component; the structure of all the different T-patterns (terminal strings); length distribution of different T-patterns both in real and randomly generated data; mean length of T-patterns; mean occurrences of T-patterns; and percent distribution of T-patterns encompassing behavioral components of hole-exploration.

#### Statistics

Concerning mean occurrences and mean durations, possible significant results were assessed using Two-Way ANOVA (strain x treatment) for independent samples, followed by Fisher’s Least Significant Difference (LSD) *post hoc* test for multiple comparisons among groups; *p* < 0.05 was considered a significant value. As to T-pattern analysis, given that each detected sequence firmly orbits around the detection of a significant relationship among the events in patterns in data sets with thousands of events, an enormous number of probable different sequences might be possible. Such an aspect raises an important issue: the possibility that T-patterns are detected by mere chance. Theme (Wageningen, Netherlands: Noldus Information Technology bv), the software tool used for T-pattern detection, deals with such an important issue by repeatedly randomizing and re-analyzing the original data, using the same search parameters utilized in the detection process performed in the real data. Then, the mean number of T-patterns of each length detected in the randomized data sets is compared with the number of patterns identified in the original data. Mean occurrences and mean length of T-patterns detected in real data were assessed using the Two-Way ANOVA for independent samples followed by Fisher’s PLSD *post hoc* test for multiple comparisons among groups; *p* < 0.05 was considered a significant value. Finally, the percent distribution of T-patterns encompassing behavioral components of hole-exploration was assessed using the Chi-square test; *p* < 0.05 was considered significant.

### Neurochemical Analyses

#### Tissue Processing for Post-mortem Analysis

We followed the previously described methodology (De Deurwaerdère et al., 2020) with some modifications (Chagraoui et al., 2019). Briefly, a different cohort of rats was deeply anesthetized using chloral hydrate and sacrificed 30 min after drug injection (see pharmacological treatment) and the brain was then quickly removed. The brains were frozen using liquid nitrogen and stored in a freezer at −80°C. The brains were cut using a cryostat at −24°C, and bilateral “punches” were made of various brain structures of interest using steel cannulae (Supplementary Figure 2). The brain regions of interest were motivated by previous results showing alterations in monoamines in brain regions involved in GAERS ASs (Yagüe et al., 2013; Crunelli and Di Giovanni, 2014). Additional brain regions, such as the dorsal hippocampus (dHP), the striatum, and the nucleus accumbens (NAc) were also included because of the involvement of the monoaminergic system in the comorbid symptoms (mood-like and cognitive-like symptoms) of GAERS. For certain structures dHP, dorsal lateral geniculate nucleus (dLGN), ventral basal thalamus [(VB) composed of the ventral posterior thalamus and the ventrolateral thalamus], a higher number of punches was collected to anticipate low levels of monoamines (Supplementary Figure 2). These tissue punches were placed in weighed 0.6-ml tubes and frozen at −80°C. On the day of the biochemical analysis, the tubes containing the samples were carefully re-weighed (Dellu-Hagedorn et al., 2017) and 100 μl of perchloric acid (HClO_4_.1N, 4°C) were added. The samples were sonicated for about 6 s and centrifuged at 13,000 rpm for 30 min at 4°C. A volume of 10 or 20 μl (depending on the brain region analyzed) of the supernatant was injected into an HPLC system.

### High-Pressure Liquid Chromatography Analysis and Electrochemical Detection

The tissue concentrations of monoamines were measured by HPLC, coupled to a coulometric detection system (Chagraoui et al., 2019). The mobile phase was composed of NaH_2_PO_4_ (70 mM), EDTA (0.1 mM), triethylamine (100 μl/l), sodium octyl sulfate (100 mg/l), and methanol (7%) diluted in de-ionized water (pH 4.2, adjusted with orthophosphoric acid) as previously reported (De Deurwaerdere et al., 1995). The mobile phase, filtered (0.22 μm) before its installation in the system, was conveyed through the HPLC column (Hypersyl, C18, 15 cm × 4.6 mm, particle size 5 μm, C.I.L.), preceded by a Brownlee-Newgard precolumn (RP-8, 15 × 3.2 mm, 7 μm; C.I.L.) using an HPLC pump (LC10Ad Vp, Shimadzu, France) at a 1.2-ml/min flow rate. The aliquots were injected using a manual injection valve (Rheodyne, model 7725i, C.I.L.) equipped with a loop of 20 μl. The elution times of the compounds were approximate as follows (in minutes): NA: 3.30; DOPAC: 4.90; DA: 6.25; 5-HIAA: 9.35; 5-HT: 16.80. The potential of the two electrodes composing the coulometric detection cell (Cell 5011, ESA, Paris, France) was fixed at +350 mV (oxidation) and −270 mV (reduction), respectively, on the electrochemical detector (CoulochemII, ESA, Paris, France). The signals from the detector were recorded on a computer through an interface (Ulyss, Azur system, Toulouse, France).

The calibration curves were adapted according to the brain areas investigated, because the quantities of monoamines and their corresponding metabolites are heterogeneous, requiring different gains set at the level of the detector using a timeline method. NA, 5-HT, and 5-HIAA contents were observed in all sampled regions. The overall sensitivity for the compounds ranged from 3 pg/10 μl for DA to 13 pg/10 μl for HVA with a signal/noise ratio of 3:1.

#### Statistical Data Analysis

The tissue levels of each molecule in the 14 brain structures were expressed in pg/mg of tissue. The ratio of DOPAC/DA and 5-HIAA/5-HT were also calculated whenever it was possible. The data are presented as the mean ± standard error of the mean (SEM).

Outlier data were discarded if they were larger than two standard deviations (SD). We addressed whether vehicle-treated NEC and GAERS had different levels of monoamines and whether the treatment of WIN 55,212-2 had distinct effects on GAERS vs. on NEC by performing a two-way ANOVA (strain x treatment). This was systematically associated with a one-way ANOVA using the group as the main factor, which was followed by the Fisher’s PLSD *post hoc* test. A similar analysis was performed for the weight of the tissue between groups for each structure (no difference in the weight of tissues was observed between the groups). In all comparisons, *p* < 0.05 was used as the criterion for significance.

## Results

### Behavioral Study

Thirty NEC and thirty GAERS rats were randomly assigned to 4 groups: NEC treated with vehicle *IP* (*n* = 15), NEC treated with 2 mg/kg *IP* of WIN 55,212-2 (*n* = 15), GAERS treated with vehicle *IP* (*n* = 15), GAERS treated with 2 mg/kg *IP* of WIN 55,212-2 (*n* = 15), and tested in hole-board arena (see Figure 1 and Supplementary Figure 1).

#### Quantitative Analysis of the Behaviors of Genetic Absence Epilepsy Rats From Strasbourg and Non-epileptic Control in the Hole-Board Occurrences

**FIGURE 2:**
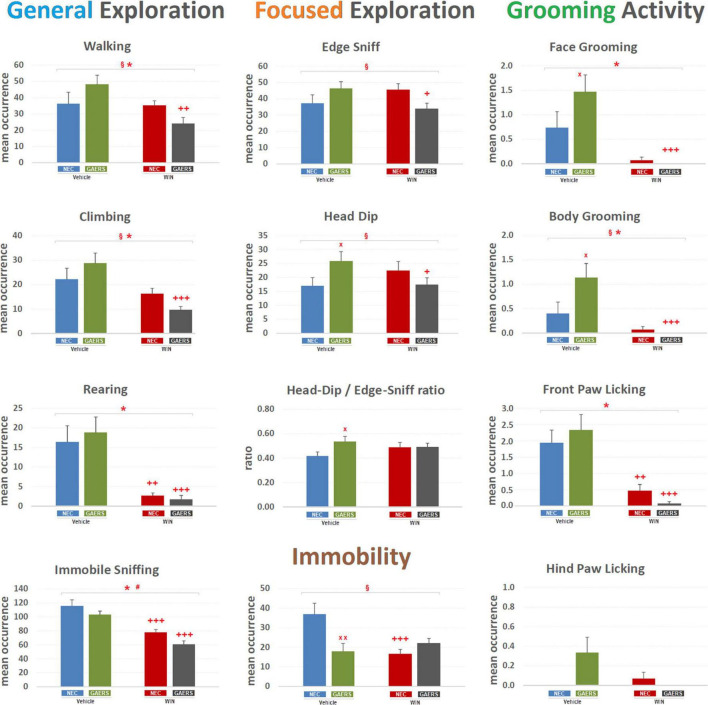
Effect of WIN 55,212-2 and its vehicle on the occurrence of different behaviors of Genetic Absence Epilepsy Rats from Strasbourg (GAERS) and non-epileptic control (NEC) rats. Administration of WIN 55,212-2 (WIN; 2 mg/kg, *IP*) and its vehicle affected the mean occurrence ± SEM of several behaviors of GAERS and NEC rats detected during the 10 min of the hole-board test. § = significant (*p* < 0.05) interaction between factors (strain x treatment, two-way ANOVA); * = significant (*p* < 0.05) main effect of pharmacological treatment (WIN vs. Vehicle, two-way ANOVA). # = significant (*p* < 0.05) effect of strain (GAERS vs. NEC, two-way ANOVA). x = significant difference between GAERS Vehicle vs. NEC Vehicle (Fisher’s LSD *post hoc* test for multiple comparisons). + = significant difference between GAERS WIN vs. GAERS Vehicle or between NEC WIN vs. NEC Vehicle (Fisher’s LSD *post hoc* test for multiple comparisons). x = *p* < 0.05, xx = *p* < 0.01; + = *p* < 0.05, ++ = *p* < 0.01 +++ = *p* < *0.001*. Data obtained from the analysis of four groups (*n* = 15 subjects in each group).

With regard to mean occurrence of different behaviors (Figure 2), two-way ANOVA (strain and treatment) revealed a significant (*p* < 0.05) main effect of WIN 55,212-2 (treatment) on General Exploration, i.e., Walking [*F*_(3_,_59)_ = 5.98; *p* < 0.05], Climbing [*F*_(3_,_59)_ = 13.73; *p* < 0.001], Rearing [*F*_(3_,_59)_ = 26.54; *p* < 0.001], Immobile-Sniffing [*F*_(3_,_59)_ = 42.26; *p* < 0.001], and Grooming Activity, i.e., Face Grooming [*F*_(3_,_59)_ = 19.27; *p* < 0.001] and Body Grooming [*F*_(3_,_59)_ = 14.93; *p* < 0.001], and Front-paw Licking [*F*_(3_,_59)_ = 32.91; *p* < 0.001]. A significant (*p* < 0.05) effect of strain (i.e., GAERS vs. NEC) was revealed only for Immobile-Sniffing [*F*_(3_,_59)_ = 5.58; *p* < 0.05]. Significant interaction between factors (i.e., strain x treatment) was revealed for General Exploration, i.e., Walking [*F*_(3_,_59)_ = 4.95; *p* < 0.05], Climbing [*F*_(3_,_59)_ = 3.83; *p* < 0.05], Focused Exploration i.e., Edge-Sniff [*F*_(3_,_59)_ = 6.08; *p* < 0.05], Head-Dip [*F*_(3_,_59)_ = 5.57; *p* < 0.05], Grooming Activity only for Body-Grooming [*F*_(3_,_59)_ = 4.42; *p* < 0.05], and Immobility [*F*_(3_,_59)_ = 9.59; *p* < 0.01]. Results of Fisher’s PLSD *post hoc* test for multiple comparisons are also illustrated in Figure 2. Interestingly, GAERS treated with vehicle showed a higher frequency of Head-Dip (*p* < 0.05), Face Grooming (*p* < 0.05), Body Grooming (*p* < 0.05), Head-Dip/Edge-Sniff ratio (*p* < 0.05), and a decreased immobility (*p* < 0.01) than vehicle-treated NEC (Figure 2). Thus, since the ratio of Head-Dip/Edge-Sniff is an indication of the difference of the anxiety status between different animal groups (Casarrubea et al., 2017, 2021b), these results further indicate that GAERS treated with vehicle are less anxious than NEC rats.

The WIN 55,212-2 significantly decreased the occurrence of Walking (*p* < 0.01), Climbing (*p* < 0.001), Rearing (*p* < 0.001), Immobile-Sniffing (*p* < 0.001), Front Paw Licking (*p* < 0.001), and Face Grooming (*p* < 0.001) and Body Grooming (*p* < 0.001), while it did not modify the occurrence of Immobility and the Head Dip/Edge-Sniff ratio (Figure 2). WIN 55,212-2 only affected a few behaviors in NEC, such as a decrease in the occurrence of Rearing (*p* < 0.01), Immobile-Sniffing (*p* < 0.001), Front Paw Licking (*p* < 0.01), and differently from its lack of effect in GAERS, it reduced the frequency of Immobility events (*p* < 0.001) (Figure 2). These results show that WIN 55,212-2 is affecting General, but not the Focused, Exploration, suggesting the main effect on locomotor behaviors.

#### Durations

**FIGURE 3:**
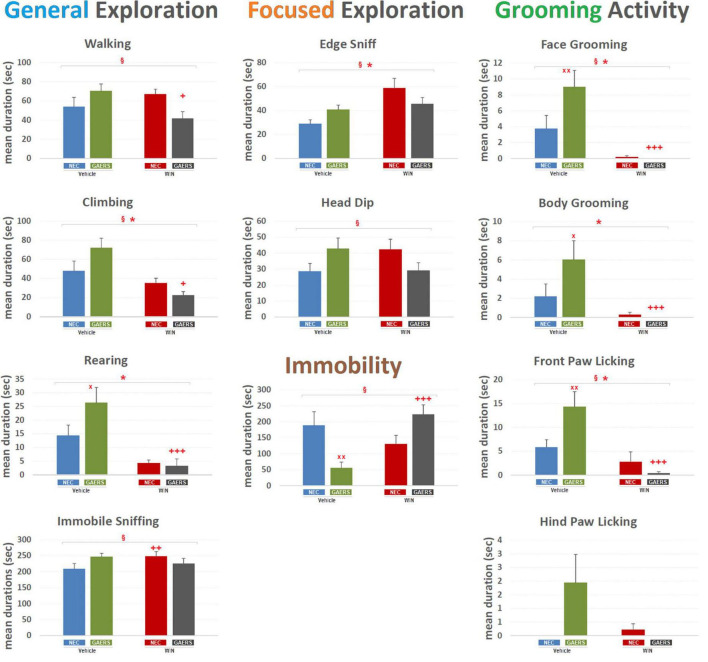
Effect of WIN 55,212-2 and its vehicle on the duration of different behaviors of GAERS and NEC rats. Administration of WIN 55,212-2 (WIN; 2 mg/kg, IP) and its vehicle affected the mean duration ± SEM of several behaviors of GAERS and NEC rats detected during the 10 min of the hole-board test. Mean durations (sec) ± SEM of all the behavioral components of the behavioral repertoire. § = significant (*p* < 0.05) interaction between factors (strain x treatment, two-way ANOVA); * = significant (*p* < 0.05) main effect of pharmacological treatment (WIN vs. Vehicle, two-way ANOVA). x = significant difference between GAERS Vehicle vs. NEC Vehicle (Fisher’s LSD *post hoc* test for multiple comparisons). + = significant difference between GAERS WIN vs. GAERS Vehicle or between NEC WIN vs. NEC Vehicle (Fisher’s LSD *post hoc* test for multiple comparisons). x = *p* < 0.05, xx = *p* < 0.01; + = *p* < 0.05, ++ = *p* < 0.01, ++ + = *p* < 0.001. Data obtained from 15 rats in each group.

As to the mean duration of the behavioral events (Figure 3), two-way ANOVA revealed a significant (*p* < 0.05) main effect of WIN 55,212-2 (treatment) for General Exploration, i.e., Climbing [*F*_(3_,_59)_ = 15.16; *p* < 0.001], Rearing [*F*_(3_,_59)_ = 20.32; *p* < 0.001], Focused Exploration only for Edge-Sniff [*F*_(3_,_59)_ = 9.45; *p* < 0.01], Grooming Activity, i.e., Front-Paw Licking [*F*_(3_,_59)_ = 16.55; *p* < 0.001], Face Grooming [*F*_(3_,_59)_ = 21.64; *p* < 0.001], and Body Grooming [*F*_(3_,_59)_ = 11.15; *p* < 0.01]. No significant effect of strain (GAERS vs. NEC) was revealed for any behavioral component. Significant interaction between factors (i.e., strain x treatment) was detected for Walking [*F*_(3_,_59)_ = 7.71; *p* < 0.01], Climbing [*F*_(3_,_59)_ = 5.34; *p* < 0.05], Immobile-Sniffing [*F*_(3_,_59)_ = 3.82; *p* < 0.05], Edge-Sniff [*F*_(3_,_59)_ = 5; *p* < 0.05], Head-Dip [*F*_(3_,_59)_ = 5.41; *p* < 0.05], Front-Paw Licking [*F*_(3_,_59)_ = 6.78; *p* < 0.05], Face-Grooming [*F*_(3_,_59)_ = 4.07; *p* < 0.05], and Immobility [*F*_(3_,_59)_ = 12.85; *p* < 0.05]. Results of Fisher’s PLSD *post hoc* test for multiple comparisons are illustrated in Figure 3. Notably, GAERS treated with vehicle showed a higher duration of Rearing (*p* < 0.05), Front Paw Licking (*p* < 0.01), Face Grooming (*p* < 0.01), Body Grooming (*p* < 0.05), and Immobility (*p* < 0.01) than vehicle-treated NEC (Figure 3). In GAERS rats, WIN 55,212-2 significantly decreased the duration of Walking (*p* < 0.05), Climbing (*p* < 0.05), Rearing (*p* < 0.001), Front Paw (*p* < 0.001) and Face Grooming Licking (*p* < 0.001), and Body Grooming (*p* < 0.001) and increase the duration of Immobility (*p* < 0.001) (Figure 3). WIN 55,212-2 affected only one behavior in NEC, i.e., it induced an increase of Immobile-Sniffing (*p* < 0.01) (Figure 3). The duration of the behavior examined further supports the occurrences showing important changes for General Exploration, but also indicates a potential effect on emotional-related behaviors (i.e., Focused Exploration and Grooming).

#### Qualitative T-Patterns Analysis of the Behaviors of Genetic Absence Epilepsy Rats From Strasbourg and Non-epileptic Control in the Hole-Bord Test

**FIGURE 4:**
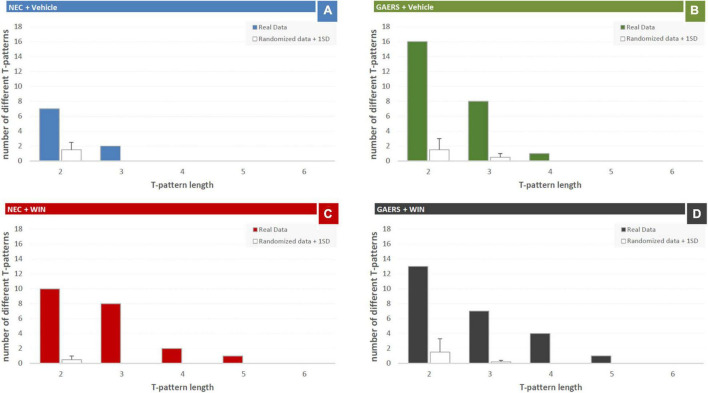
Effect of WIN 55,212-2 and its vehicle on the temporal patterns (T-patterns) in GAERS and NEC rats. Administration of WIN 55,212-2 (WIN; 2 mg/kg, *IP*) and its vehicle affected the T-pattern of behaviors detected in GAERS and NEC rats during the 10 min of the hole-board test. Numbers on the right of each string indicate their overall length and occurrences (Occs). Behavioral events in brackets do represent the textual representations of T-patterns in terms of order of events (by reading each string from left to right). Brackets do indicate hierarchical detections of events within each T-patterns on the basis of bottom-up detection process (see section “Data Analysis” for details). Numbers on the right of each string indicate the number of events (length) and the occurrences (Occs) of each T-pattern. Data were obtained from the analysis of four groups (*n* = 15 rats in each group). For abbreviations see text and Supplementary Figure 1.

**FIGURE 5:**
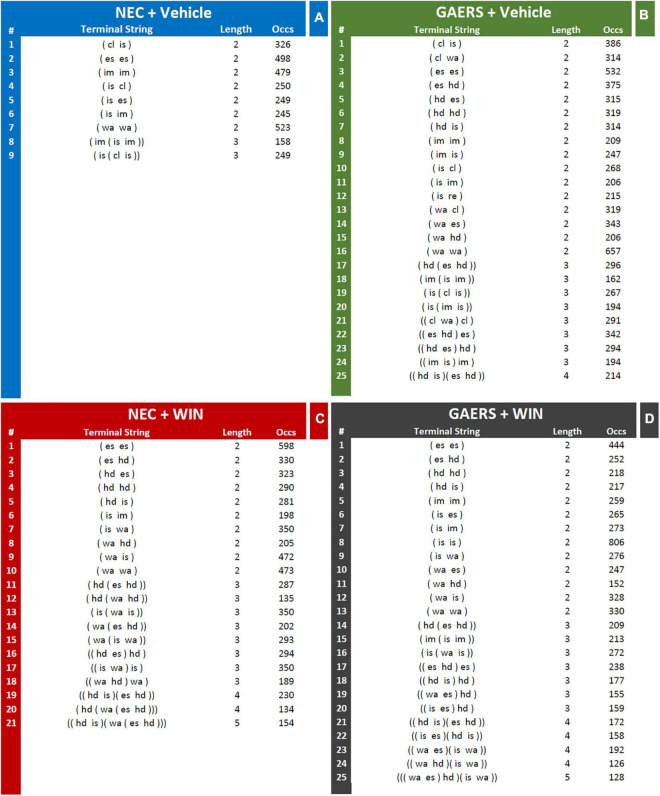
Effect of WIN 55,212-2 and its vehicle on the number of T-patterns of different lengths in GAERS and NEC rats. Administration of WIN 55,212-2 (WIN; 2 mg/kg, IP) and its vehicle affected the overall number of different T-patterns detected based on their length for each group during the 10 min of the hole-board test. Filled bars = number of patterns detected in the real data; white bars = average number + 1xSD of patterns detected in randomized data.

Results of T-pattern detection in terms of terminal strings, length, and overall occurrences are presented in Figure 3. In vehicle-treated NEC rats (Figure 4A), 9 different T-patterns occurred 2,977 times; in the GAERS treated with vehicle (Figure 4B), 25 different T-patterns occurred 7,479 times; in the NEC treated with WIN 55,212-2 (Figure 4C), 21 different T-patterns occurred 6,138 times, and in GAERS treated with WIN 55,212-2 (Figure 4D), 25 different T-patterns occurred 6,266 times. The length distribution of T-patterns (that is, the number of T-patterns of different lengths detected for each group) is illustrated in Figure 5. In NEC treated with vehicle (Figure 5A), 7 different T-patterns encompass two events and 2 contain three events; in GAERS treated with vehicle (Figure 5B), 16 T-patterns contain two events, 8 patterns three events and 1 pattern consists of four events; in NEC treated with WIN 55,212-2 (Figure 5C), 10 patterns have two events, 8 patterns three, 2 patterns four, and only 1 T-pattern has five events; and in GAERS treated with WIN 55,212-2 (Figure 5D), 13 patterns contain two events, 7 patterns three, 4 patterns four, and 1 T-pattern encompasses five events.

**FIGURE 6:**
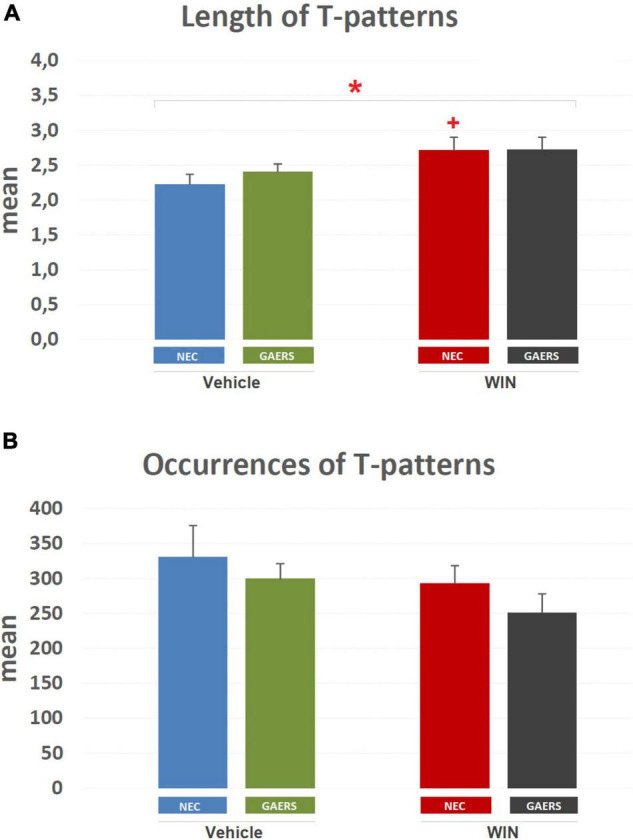
Effect of WIN 55,212-2 and its vehicle on the length and occurrences of T-patterns in GAERS and NEC rats. Administration of WIN 55,212-2 (WIN; 2 mg/kg, IP) and its vehicle affected the mean length ± SEM **(A)** and mean occurrences ± SE **(B)** of T-patterns in each group detected during the 10 min of the hole-board test. * = significant (*p* < 0.05) main effect of pharmacological treatment (Win vs. Vehicle, two-way ANOVA). Data were obtained from the analysis of four groups (*n* = 15 subjects in each group).

The mean length of T-patterns and their mean occurrence are illustrated in Figure 6. Considering mean length (Figure 6A), two-way ANOVA (strain vs. treatment) revealed a significant (*p* < 0.05) main effect of the pharmacological treatment (i.e., WIN 55,212-2 vs. vehicle). Results of Fisher’s LSD *post hoc* test for multiple comparisons showed a significant increase in mean length of T-patterns detected in WIN 55,212-2-treated compared to vehicle-treated NEC rats. No significant difference was detected between WIN 55,212-2-treated and vehicle-treated GAERS rats. Moreover, no significant interaction between factors was found. As to the mean occurrence of T-patterns (Figure 6B), no significant difference was detected by two-way ANOVA.

**FIGURE 7:**
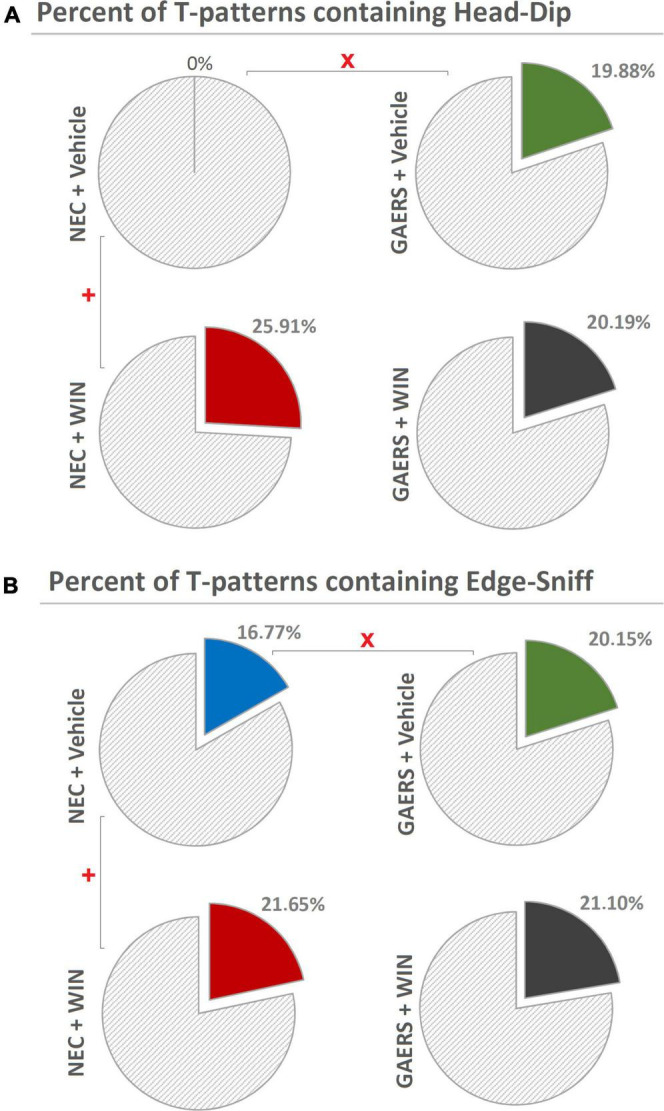
Effect of WIN 55,212-2 and its vehicle on the percent distribution of T-patterns containing Head-Dip and Edge-Sniff in GAERS and NEC rats. Administration of WIN 55,212-2 (WIN; 2 mg/kg, IP) and its vehicle affected the percent distribution of T-patterns containing Head-Dip **(A)** and Edge-Sniff **(B)** in their sequences during the 10 min of the hole-board test. x = significant (*p* < 0.05) difference between GAERS Vehicle vs. NEC Vehicle (Chi-Square test). + = significant (*p* < 0.05) difference between GAERS WIN vs. GAERS Vehicle or between NEC WIN vs. NEC Vehicle (Chi-Square test).

Finally, the percent distributions of T-patterns encompassing behavioral components of hole-exploration predictive of the animal anxiety level (Casarrubea et al., 2021b) are illustrated in Figure 7. Chi-square test revealed significant (*p* < 0.05) differences between vehicle-treated GAERS and vehicle-treated NEC, WIN 55,212-2-treated NEC, and vehicle-treated NEC, both for T-patterns encompassing Head-Dip (Figure 7A) and Edge-Sniff (Figure 7B), but no significant differences between vehicle- and WIN 55,212-2-treated GAERS in both parameters (Figures 7A,B).

### Neurochemical Study

Compelling evidence shows that ECS interaction with monoaminergic systems is strongly involved in many psychiatric disorders including anxiety (Colangeli et al., 2021; Mendiguren et al., 2021). We, therefore, studied the effect of WIN 55,212-2 treatment (2 mg/kg, *IP*) and its vehicle on the levels of monoamines and their metabolites in 14 brain areas of GAERS (*n* = 12) and NEC (*n* = 12) rats. The position of the punches used to extract the tissue samples from the cortex (M1, S1, and V1), NAc core (Co), striatum (mS, lS) thalamus (AD, NRT, VB, Po, and dLGN), dHP, EPN, and SN is shown in Supplementary Figure 2.

#### Monoamine Levels in Genetic Absence Epilepsy Rats From Strasbourg and Non-epileptic Control Rats Treated With WIN 55,212-2 and Its Vehicle

The analysis revealed both strain and drug effects, which are selective for each monoamine.

A significant strain x treatment interaction on NA tissue levels was observed only in the EPN (Table 1). Results of Fisher’s PLSD *post hoc* test show that NA is decreased in GAERS treated with vehicle (*p* < 0.001) and WIN 55,212-2 enhanced NA tissue levels in the EPN of GAERS (*p* < 0.001) compared to NEC.

**TABLE 1:** Effect of WIN 55,212-2 and its vehicle on Noradrenaline (NA) levels in selected brain areas of Genetic Absence Epilepsy Rats from Strasbourg (GAERS) and non-epileptic control (NEC) rats.

NA	Vehicle		WIN 55,212-2		ANOVA
	NEC	GAERS	NEC	GAERS	
**Cortex**					
M1	177 ± 26	157 ± 11	148 ± 18	229 ± 54	*F(1,17) = 2.6*
S1	208 ± 28	175 ± 9	206 ± 33	156 ± 6	*F(1,20) = 0.15*
V1	143 ± 17	192 ± 6	216 ± 40	247 ± 38	*F(1,20) = 0.02*
**Thalamus**					
AD	133 ± 67	177 ± 38	199 ± 54	301 ± 77	*F(1,19)* = *0.03*
dLGN	198 ± 25	223 ± 51	138 ± 12	147 ± 29	*F(1,19)* = *0.05*
NRT	141 ± 38	259 ± 59	257 ± 51	280 ± 31	*F(1,18)* = *1*
Po	375 ± 128	324 ± 52	320 ± 43	387 ± 54	*F(1,15)* = *0.5*
VB	136 ± 16	150 ± 15	131 ± 27	114 ± 24	*F(1,20)* = *0.5*
**Basal Ganglia**					
NAc (core)	102 ± 11	88 ± 15	112 ± 41	200 ± 49	*F(1,20)* = *2.3*
mSTR	61 ± 11	60 ± 19	58 ± 18	69 ± 19	*F(1,20)* = *0.13*
lSTR	72 ± 4	57 ± 4	62 ± 8	66 ± 5	*F(1,19)* = *3.1*
EPN	301 ± 15	147 ± 16***	230 ± 58	293 ± 34^###^	*F(1,20)* = *9.6^^*
SN	154 ± 26	126 ± 30	259 ± 47	224 ± 34	*F(1,20)* = *0.01*
**Hippocampus**					
dH	256 ± 20	164 ± 36	212 ± 30	192 ± 27	*F(1,20)* = *1.6*

Administration of WIN 55,212-2 (WIN; 2 mg/kg, IP) and its vehicle affected noradrenaline (NA) levels in selected brain areas of GAERS and NEC rats. NA levels (pg/mg tissue) are expressed as mean ± SEM. The ANOVA column shows the results of the two-way ANOVA (WIN x strain; ^^p < 0.01). ***p < 0.001 with respect to NEC vehicle; ^###^p < 0.001 with respect to GAERS vehicle (PLSD test after significant one-way ANOVA). AD, anterodorsal nucleus of the thalamus; dHP, dorsal part of the hippocampus; EPN, entodepuncular nucleus; dLGN, dorsal lateral geniculate nucleus; lSTR and mSTR, lateral and medial striatum; M1, motor cortex; NAc, nucleus accumbens; NRT, nucleus reticularis thalami; Po, posterior thalamic nucleus; S1, somatosensorial cortex (posterior part); SN, substantia nigra; VB, ventrobasal complex of the thalamus; V1, visual cortex.

A significant strain x treatment interaction on DA tissue content was observed in the dLGN only (Table 2A). Fisher’s PLSD *post hoc* test revealed that DA levels in the dLGN were higher in vehicle-treated GAERS than in control NEC (*p* < 0.05). The DOPAC/DA ratio was increased for vehicle-treated GAERS than in control NEC (*p* < 0.05). WIN 55,212-2 reduced DA tissue content in the dLGN of GAERS but not in NEC (*p* < 0.05). The effect of WIN 55,212-2 on the DOPAC/DA ratio was modified in the NAc (Co) only (*p* < 0.05) (Table 2B), where the ratio was significantly decreased in GAERS only (*p* < 0.05). The full analysis of the effect of WIN 55,212-2 on DOPAC or HVA tissue content (not shown) did not reveal any strain x treatment interaction.

**TABLE 2:** Effect of WIN 55,212-2 and its vehicle on Dopamine (DA) tissue content and the ratio DOPAC/DA in selected brain regions of GAERS and NEC rats.

A. DA	Vehicle		WIN 55,212-2		ANOVA
	NEC	GAERS	NEC	GAERS	
**Cortex**					
M1	10 ± 2	11 ± 5	16 ± 5	66 ± 34	*F(1,16)* = *2.7*
S1 (PSC)	14 ± 3	20 ± 7	16 ± 4	16 ± 2	*F(1,19)* = *0.37*
V1	9 ± 1.5	16 ± 1.7	9 ± 1.6	18 ± 3.1	*F(1,17)* = *0.3*
**Thalamus**					
AD	22 ± 5.5	22 ± 3	31 ± 3.7	32 ± 6.8	*F(1,17)* = *0.13*
dLGN	34 ± 7.5	68 ± 17*	55 ± 9.5	36 ± 6.5^#^	*F(1,18)* = *5^*
Rt	56 ± 12	33 ± 7	34 ± 8	23 ± 2	*F(1,14)* = *0.53*
Po	82 ± 19	59 ± 16	74 ± 12	37 ± 16	*F(1,17)* = *3*
VB	42 ± 7	23 ± 2	29 ± 9	34 ± 13	*F(1,19)* = *1.7*
**Basal Ganglia**					
NAc (core)	2834 ± 566	2684 ± 473	2105 ± 203	2939 ± 728	*F(1,18)* = *0.73*
Medial STR	2268 ± 446	2775 ± 647	2678 ± 388	4261 ± 1153	*F(1,20)* = *0.58*
Lateral STR	5025 ± 414	4959 ± 1042	4763 ± 1021	5534 ± 935	*F(1,20)* = *0.22*
EPN	100 ± 29	100 ± 17	144 ± 41	118 ± 29	*F(1,19)* = *0.07*
SN	280 ± 91	243 ± 81	354 ± 44	384 ± 64	*F(1,20)* = *0.21*
**Hipp.**					
dH	4.1 ± 1.6	3.3 ± 0.9	2.2 ± 0.3	2.2 ± 0.8	*F(1,16)* = *0.004*

**B. DOPAC/DA**					

**Cortex**					
M1	*nd*	*nd*	*nd*	*nd*	
S1	0.64 ± 0.08	1.2 ± 0.47	0.73 ± 0.18	1.06 ± 0.28	*F(1,17)* = *0.16*
V1	0.76 ± 0.23	0.94 ± 0.13	1.28 ± 0.54	0.63 ± 0.18	*F(1,14)* = *1.4*
**Thalamus**					
AD	1.08 ± 0.5	0.58 ± 0.15	0.86 ± 0.24	0.36 ± 0.06	*nd*
dLGN	0.91 ± 0.17	0.89 ± 0.22	0.38 ± 0.05	0.96 ± 0.09	*F(1,18)* = *3.8*
NRT	0.41 ± 0.16	1.03 ± 0.44	1.12 ± 0.15	0.83 ± 0.18	*nd*
Po	*nd*	*nd*	*nd*	*nd*	
VB	0.35 ± 0.13	0.32 ± 0.08	0.41 ± 0.23	0.15 ± 0.04	*F(1,18)* = *1.5*
**Basal Ganglia**					
NAc (core)	0.39 ± 0.02	0.56 ± 0.05*	0.45 ± 0.05	0.4 ± 0.03^#^	*F(1,18)* = *6.1^*
mSTR	0.2 ± 0.02	0.26 ± 0.03	0.2 ± 0.02	0.26 ± 0.07	*F(1,20)* = *0.001*
lSTR	0.28 ± 0.06	0.22 ± 0.03	0.22 ± 0.01	0.21 ± 0.01	*F(1,20)* = *0.55*
EPN	0.34 ± 0.08	0.27 ± 0.06	0.33 ± 0.09	0.49 ± 0.08	*F(1,19)* = *2*
SN	1.37 ± 0.17	1.42 ± 0.17	1.33 ± 0.14	1.3 ± 0.25	*F(1,20)* = *0.04*
**Hipp.**					
dH	*nd*	*nd*	*nd*	*nd*	

The results correspond to the mean ± SEM of DA contents in **A** (pg/mg tissue) and the ratio DOPAC/DA in **B** in various brain regions. The ANOVA column reports the results of the two-way ANOVA (WIN x genotype; ^p < 0.05). *p < 0.05 with respect to NEC vehicle; ^#^p < 0.05 with respect to GAERS vehicle (PLSD test after significant one-way ANOVA). nd, not determined due to technical problems. AD, anterodorsal nucleus of the thalamus; dHP, dorsal part of the hippocampus; EPN, entodepuncular nucleus; dLGN, dorsal lateral geniculate nucleus; lSTR and mSTR, lateral and medial striatum; M1, motor cortex; NAc, nucleus accumbens; NRT, nucleus reticularis thalami; Po, posterior thalamic nucleus; S1, somatosensorial cortex (posterior part); SN, substantia nigra; VB, ventrobasal complex of the thalamus; V1, visual cortex.

The effect of treatments on 5-HT levels is reported in Table 3. Fisher’s PLSD *post hoc* test shows that vehicle-treated GAERS showed lower 5-HT tissue contents in M1, SN, and dHP (*p* < 0.05). In these regions, WIN 55,212-2 instead increased 5-HT tissue contents in M1 (p.05), SN (*p* < 0.001) GAERS compared to NEC rats. Similarly, WIN 55,212-2 differentially altered 5-HIAA content in GAERS and NEC in M1 [two-way ANOVA, gene x treatment, *F*(1,20) = 10.3, *p* < 0.01], in LGN [*F*(1,20) = 5.6, *p* < 0.05], and in dHP [*F*(1,20) = 8.9, *p* < 0.01] (not shown). In most cases, the effects of WIN 55,212-2 on 5-HT and 5-HIAA were similar and the 5-HIAA/5-HT ratio increased only V1 (*p* < 0.001) (Table 3B).

**TABLE 3:** Effect of WIN 55,212-2 and its vehicle on 5-HT tissue content and the ratio 5-HIAA/5-HT in selected brain regions of GAERS and NEC rats.

A. 5-HT	Vehicle		WIN 55,212-2		ANOVA
	NEC	GAERS	NEC	GAERS	
**Cortex**					
M1	214 ± 60	95 ± 12*	99 ± 11	215 ± 54^#^	*F(1,20)* = *5.9****^***
S1 (PSC)	80 ± 14	55 ± 6	80 ± 11	66 ± 11	*F(1,20)* = *0.29*
V1	46 ± 12	62 ± 11	71 ± 17	63 ± 15	*F(1,20)* = *0.7*
**Thalamus**					
AD	76 ± 14	75 ± 11	82 ± 12	144 ± 34	*F(1,18)* = *2.2*
dLGN	197 ± 41	180 ± 48	125 ± 10	134 ± 19	*F(1,19)* = *0.17*
NRT	114 ± 31	77 ± 24	142 ± 39	150 ± 31	*F(1,18)* = *0.45*
Po	278 ± 71	282 ± 53	284 ± 39	449 ± 135	*F(1,20)* = *0.93*
VB	66 ± 16	103 ± 17	74 ± 39	115 ± 44	*F(1,17)* = *0.1*
**Basal Ganglia**					
NAc (core)	363 ± 60	282 ± 47	328 ± 78	369 ± 60	*F(1,20)* = *0.94*
Medial STR	244 ± 57	226 ± 58	166 ± 34	180 ± 72	*F(1,18)* = *0.06*
Lateral STR	249 ± 19	207 ± 47	211 ± 41	276 ± 57	*F(1,20)* = *1.5*
EPN	203 ± 21	200 ± 37	185 ± 35	240 ± 20	*F(1,20)* = *0.94*
SN	966 ± 162	477 ± 125*	1089 ± 112	1429 ± 267^###^	*F(1,20)* = *5.5****^***
**Hipp.**					
dHP	170 ± 10	105 ± 13*	117 ± 15	165 ± 35	*F(1,19)* = *5.2****^***

**B. DOPAC/DA**					

**Cortex**					
M1	1.67 ± 0.31	2.01 ± 0.27	1.74 ± 0.13	2.39 ± 0.28	*F(1,20)* = *0.32*
S1 (PSC)	2.32 ± 0.35	2.29 ± 0.41	1.81 ± 0.28	2.06 ± 0.31	*F(1,20)* = *0.04*
V1	2.16 ± 0.25	1.79 ± 0.11	2.13 ± 0.24	3.37 ± 0.37^###^	*F(1,18)* = *6.1****^***
**Thalamus**					
AD	3.26 ± 0.42	4.01 ± 0.63	2.25 ± 0.23	2.77 ± 0.46	*F(1,18)* = *0.2*
dLGN	1.54 ± 0.36	1.07 ± 0.17	1.16 ± 0.26	0.88 ± 0.13	*F(1,17)* = *0.5*
NRT	1.24 ± 0.38	2.08 ± 0.39	1.34 ± 0.2	1.4 ± 0.23	*F(1,17)* = *1.6*
Po	1.15 ± 0.44	1.54 ± 0.51	0.62 ± 0.14	1.32 ± 0.41	*F(1,19)* = *0.13*
VB	2.61 ± 0.62	1.84 ± 0.33	2.09 ± 0.29	1.75 ± 0.34	*F(1,17)* = *0.25*
**Basal Ganglia**					
NAc (core)	0.9 ± 0.09	1.18 ± 0.14	1.48 ± 0.5	1.44 ± 0.32	*F(1,20)* = *0.22*
Medial STR	0.86 ± 0.08	1.17 ± 0.11	1.04 ± 0.17	1.65 ± 0.23	*F(1,17)* = *1.16*
Lateral STR	1.18 ± 0.17	1.53 ± 0.26	1.14 ± 0.17	1.26 ± 0.19	*F(1,20)* = *0.3*
EPN	1.45 ± 0.26	1.72 ± 0.32	2.02 ± 0.22	1.95 ± 0.24	*F(1,20)* = *0.41*
SN	0.64 ± 0.06	0.72 ± 0.09	0.62 ± 0.05	0.63 ± 0.08	*F(1,20)* = *0.26*
**Hipp.**					
dHP	3.26 ± 0.29	3.12 ± 0.33	3.2 ± 0.42	3.11 ± 0.51	*F(1,20)* = *0.1*

The results correspond to the mean ± SEM of 5-HT contents (pg/mg tissue) in **A** and the ratio 5-HIAA/5-HT in **B** in various brain regions. The ANOVA column reports the results of the two-way ANOVA (WIN x genotype; ^p < 0.05). *p < 0.05 with respect to NEC vehicle; ^#^p < 0.05, ^###^p < 0.001 with respect to GAERS vehicle (PLSD test after significant one-way ANOVA). Of note, the two way ANOVA (strain x treatment) revealed significant modifications for the 5-HIAA content in the M1 [F(1,20) = 10.3, p < 0.01], the dLGN [F(1,20) = 5;58, p < 0.05], and the dHP [F(1,19) = 8.9, p < 0.01] (see Section “Results”). AD, anterodorsal nucleus of the thalamus; dHP, dorsal part of the hippocampus; EPN, entodepuncular nucleus; dLGN, dorsal lateral geniculate nucleus; lSTR and mSTR, lateral and medial striatum; M1, motor cortex; NAc, nucleus accumbens; NRT, nucleus reticularis thalami; Po, posterior thalamic nucleus; S1, somatosensorial cortex (posterior part); SN, substantia nigra; VB, ventrobasal complex of the thalamus; V1, visual cortex.

## Discussion

In the present study, we show that vehicle-treated GAERS were more immobile and less anxious and had lower 5-HT levels in the cortex, dHP, and SN compared to vehicle-injected NEC. Moreover, acute systemic administration of 2 mg/kg of the CB1/2R agonist WIN 55,212-2 (Howlett et al., 2002) induced strain-dependent effects both in anxiety/motor behavior and monoamine levels in GAERS and NEC rats. Such traits of the two inbred strains after WIN 55,212-2 treatment and in control conditions have not yet been described and confirm an alteration of the ECS already observed in GAERS (Roebuck et al., 2021) and WAG/Rij rats (van Rijn et al., 2010). Another important finding of this study is that multivariate T-pattern analysis is more sensitive than classical quantitative analysis in determining neophilia changes as assessed in the hole-board.

### Distinct Reactivity of Genetic Absence Epilepsy Rats From Strasbourg and Non-epileptic Control to Vehicle Administration

Mixed results have been reported on the anxiety levels of naïve GAERS rats from the original colony in Strasburg showing GAERS being less anxious than Wistars on the open field tests (OFT) (Studer et al., 2019) and the elevated plus-maze (EPM) tests (Marques-Carneiro et al., 2014), or equally anxious to NEC (Vergnes et al., 1991). On the other hand, heightened anxiety levels have been consistently observed in the GAERS from the colony established in Melbourne and Saskatoon (the latter derived from the former one) (Jones et al., 2008, 2010; Dezsi et al., 2013; Powell et al., 2014; Marks et al., 2016, 2019). When we analyzed the data obtained in vehicle-injected rats, we found that NEC appeared to be more anxious than GAERS on the hole-board task, a finding that has not been documented previously in drug-naïve GAERS and NEC (Jones et al., 2008, 2010; Dezsi et al., 2013; Powell et al., 2014; Marks et al., 2016, 2019). From the quantitative analysis of the hole-board data, we did observe several behavioral changes among these two inbred strains treated with vehicle. GAERS had a higher occurrence and duration of Head-Dip, grooming, and lower frequency and duration of immobility events and rearing duration. On the other hand, climbing behavior (both frequency and duration) during the test was not different from NEC. Moreover, the Head-Dip/Edge-Sniff ratio was also increased in GAERS compared to NEC. The reduced anxiety-like phenotype with neophilic responses observed in vehicle-treated GAERS compared to NEC is consistent with the mixed quantitative-qualitative analysis of the T-patterns detected. Indeed, vehicle-treated NEC rats had a much lower number and less complex T-pattern sequences compared to vehicle-treated GAERS, a condition that has been related to a lower anxious status (Casarrubea et al., 2021b). Notably, vehicle-treated NEC rat’s structural behavioral analysis did not show any T-patterns containing head-dips and only a few containing edge-sniffs. On the other hand, in GAERS rats treated with the vehicle, the T-pattern variability was greater as was its complexity and shows T-patterns containing Head-Dips. GAERS rats, therefore, are motivated to explore more by implementing more complex and articulated strategies, in line with lower anxiety levels compared to vehicle-treated NEC (Casarrubea et al., 2021b).

This is the first time that GAERS are reported to be less anxious than NEC, but we need to underline that our animals were treated with the vehicle and were not untouched like those in previous studies (Jones et al., 2008, 2010; Dezsi et al., 2013; Powell et al., 2014; Marks et al., 2016, 2019). Indeed, when a vehicle was administered, GAERS rats from the Saskatoon colony no longer exhibited significant differences in anxiety-like behavior compared to NEC (Marks et al., 2019; Roebuck, 2021). The leveling shown by these studies (Marks et al., 2019; Roebuck, 2021) and our present evidence, of the anxiety status between GAERS and NEC treated with the vehicle compared to naïve animals, is puzzling. It might be explained taking into account that restraining and contextual drug administration are indeed capable to increase stress and affect the response of animals to behavioral tests (Stuart and Robinson, 2015). Stress may have resulted in the suppression of spike and wave discharges (SWDs) (Tolmacheva et al., 2012) since the number of SWDs directly correlates with anxiety (Shaw et al., 2009). Nevertheless, this effect of stress on reducing SWDs and anxiety-like behavior is unlikely to be responsible for this scenario since our preliminary data show that naïve untouched NEC rats from our colony are less anxious than naïve GAERS (Di Giovanni, unpublished observation), consistently with previous evidence from other colonies (Jones et al., 2008, 2010; Dezsi et al., 2013; Powell et al., 2014; Marks et al., 2016, 2019). Thus, a more possible alternative is that the emotional and locomotor responses in GAERS are wrongly interpreted as a sign of anxiolysis because vehicle-treated NEC rats are in reality more anxious than normal untouched NEC due to a higher sensitivity to the anxiogenic injection procedure compared to GAERS. Indeed, we observed low mobility in vehicle-treated NEC that may be related to an increase in stress in this control strain. Moreover, higher tissue levels of 5-HT in the dHP, which have been linked to heightened anxiety (File et al., 2000; Barnes et al., 2021; Villas-Boas et al., 2021), were observed in vehicle-treated NEC rats compared to GAERS. Consistent with this hypothesis, inbred non-epileptic August × Copenhagen-Irish (ACI) control rats showed a higher reactivity of the hypothalamic-pituitary-adrenal (HPA) axis to acute stress than the epileptic inbred WAG/Rij rats (Tolmacheva et al., 2012). Our findings, therefore, add further evidence to the previous data showing that the NEC rats, inbred to be selectively resistant to SWDs (Vergnes et al., 1982; Marescaux et al., 1992; Depaulis et al., 2016), exhibit distinct anatomy, physiology, and pharmacology than normal outbred Wistar rats (Marques-Carneiro et al., 2014; Powell et al., 2014; Fauvelle et al., 2015; Bombardi et al., 2018).

In terms of monoamine levels in brain areas involved in the generation of SWDs, such as the thalamus (McCafferty et al., 2018; Crunelli et al., 2020), we observed a change only limited to the dLGN. Thus, vehicle-treated GAERS dLGN showed more than double DA content compared to NEC. Considering that the release of DA in the thalamus is associated with arousal levels, visual attention, and stimulus novelty (McCormick, 1992), one hypothesis regarding this finding may be that the increased DA levels in dLGN leading to a heightened level of sensitivity are linked to high emotionality and motor activation of GAERS, although further investigation of this possibility is required.

Our findings in the basal ganglia are also intriguing. Basal ganglia play a pivotal role in ASs, classically considered modulatory (Depaulis et al., 1994; Deransart and Depaulis, 2002) and recently shown to be necessary for the generation and maintenance of SWDs (Miyamoto et al., 2019). The decrease in NA in the EPN and 5-HT in the SN of GAERS could be an endogenous protection mechanism against the generation of ASs. These monoamines decrease the firing of GABAergic basal ganglia output neurons inhibiting ASs both indirectly *via* the nigra/superior colliculus/thalamic projection (Deransart and Depaulis, 2002) and directly *via* the Nigro-thalamic pathway (Paz et al., 2007). The decrease in basal ganglia output would lead to a decrease in GABA release in the thalamus that might be a compensation for the excessive tonic GABA_*A*_ receptor inhibition present in GAERS rats (Cope et al., 2009). Moreover, the decrease in basal ganglia output would also be consistent with the higher mobility (Freeze et al., 2013) of GAERS compared to NEC rats.

In summary, the neurochemical changes that we found in GAERS and NEC rats, are similar to those described in WAG/Rij (Midzyanovskaya et al., 2020), another CAE model (Coenen and Van Luijtelaar, 2003) and ACI rats, respectively, and they are consistent with our behavioral results.

### Distinct Responses of Genetic Absence Epilepsy Rats From Strasbourg vs. Non-epileptic Control to WIN 55,212-2 Administration

The WIN 55,212-2 caused strain-dependent effects for most of the behaviors examined in the hole-board test by standard quantitative analysis, with an increase and a reduction of the anxiety-like behavior in GAERS and NEC, respectively. Notably, the pan-T-type calcium channel antagonist Z944 was shown to reduce anxiety-like behavior in NEC and increased it in GAERS rats (Marks et al., 2019), and both eCBs and phytocannabinoids can directly inhibit T-type calcium channels (Chemin et al., 2001; Ross et al., 2008). Thus, it would be interesting to know whether the effects of the synthetic cannabinoid WIN 55,212-2 observed here also depend on the modulation of this subtype of calcium channels. Our findings, together with the evidence showing that the CB1R positive allosteric modulator GAT211 failed to induce any changes in GAERS and NEC anxiety-like behaviors and motor activity in other anxiety tests (Roebuck, 2021), suggest that the control of anxiety/motor response by the eCBs in GAERS and NEC is strain-dependent and evident when phasically activated.

Here, we observed that GAERS rats showed a neophobic response and were much less prone to explore (i.e., reduction of both general and focused exploration), or self-groom and they were more immobile after administration of WIN 55,212-2, in comparison to NEC. On the other hand, WIN 55,212-2 administration failed to alter the duration of either general exploration (apart from reducing rearing occurrences) focused hole exploration, or grooming/licking activity, and instead reduced immobility frequency (but not its duration) in NEC. While the effect of WIN 55,212-2 on motor behavior is quite clear from the quantitative analysis, with clear sedation in GAERS and no effect in NEC, a more cautious interpretation of its effect on the anxiety status is necessary. Indeed, the decrease in Head-Dipping and edge-sniffing behaviors because of WIN 55,212-2 might have been confounded by the strong sedative effect of CB1R activation in GAERS. Indeed, it has been reported that Head-Dipping and locomotion are highly correlated (Kliethermes and Crabbe, 2006).

Because of the strength of the multivariate T-pattern analysis (Casarrubea et al., 2015, 2020; Aiello et al., 2020), we were able to reveal hidden behavioral features and, thus, differentiate WIN 55,212-2’s effect on anxiety-like behavior from its sedative effect. T-pattern analysis can, indeed, reveal a different scenario from quantitative evaluations of individual parameters, separate from the comprehensive structure of the behavior that can be misleading in evaluating the anxiety status of an animal (Casarrubea et al., 2017, 2021a,b), especially in conditions of contextual motor impairment. The administration of WIN 55,212-2 did not modify the variability and complexity of the behavior of the GAERS, but it did in the NEC rats compared to their respective vehicle groups. In this regard, the number and the average length of the T-patterns were unchanged in GAERS but increased in NEC by WIN 55,212-2. Furthermore, the percentage of the T-patterns containing Head-Dip and Edge-Sniff were similar in both GAERS rats treated with vehicle and those treated with WIN 55,212-2, suggesting a lack of effect on anxiety-like behavior. An opposite result was observed in NEC, where WIN 55,212-2 produced a remarkable appearance of the T-patterns containing Head-Dip and an increase of those containing Edge-Sniff, a scenario compatible with a heightened state of anxiety in these animals and an anxiolytic drug effect (Casarrubea et al., 2015, 2017, 2018, 2020, 2021b). GAERS and NEC rats treated with WIN 55,212-2 did not differ for T-patterns containing Head-dip and Edge-Sniff, indicating that the two strains had a similar anxiety status after treatment. This further supports the evidence from the quantitative analysis showing that the Head-Dip/Edge-Sniff ratio of their frequencies, which is known to be an indication of the difference in anxiety-like behavior between two experimental groups (Casarrubea et al., 2021b) [for example, higher in rats treated with diazepam (Casarrubea et al., 2009b)], did not vary between the drug-treated GAERS and NEC. Thus, after WIN 55,212-2 treatment, the two inbred rat strains displayed similar levels of neophilia/neophobia and exploratory behavior, flattening the behavioral differences seen instead in the controls between GAERS and NEC (see next section) when evaluated in the new environment of the hole board. The inclusion of a second control, such as a Wistar group, will be necessary to answer the question of whether WIN 55,212,2 treated GAERS and NEC were in a high or low level of anxiety.

The ECS plays a complex role in the regulation of emotional states and involves interaction with monoamines (Van Bockstaele, 2012; Colangeli et al., 2021). Moreover, cannabinoid induces anxiolysis in previously stressed animals (Campos et al., 2010; Iffland and Grotenhermen, 2017) and the anxiety responses and exploration behavior strictly depend on the animal’s level of stress (Campos et al., 2010). Therefore, NEC rats might have an anxiolytic response to WIN 55,212-2 because of their higher stress reactivity. This hypothesis, supported by the evidence that NEC treated with the vehicle are more anxious than GAERS, warrants further investigation. A possible explanation for the strain-dependent effect on the anxious state elicited by WIN 55,212-2 is an alteration of the ECS, which has already been shown in other rat strains (Arnold, 2000; Arnold et al., 2001, 2010). Indeed, Roebuck et al. (2021) have recently shown that compared to NEC, GAERS have reduced cortical and hippocampal CB1R levels and increased levels of 2-arachidonoylglycerol (2-AG) in the cortex and hippocampus, and both anandamide and 2-AG in the cerebellum.

The effects induced by the activation of CB1Rs by WIN 55,212-2 on the anxiety status of the animals are likely to involve monoamines, such as NA, DA, and 5-HT (Colangeli et al., 2021; Mendiguren et al., 2021). Our neurochemical results show some strain-dependent change also in the levels of monoamines induced by WIN 55,212-2. Indeed, in some brain regions, WIN 55,212-2 induced opposite changes in GAERS compared to NEC and “normalized” the values to those of vehicle-treated NEC rats. Thus, WIN 55,212-2 increased NA in the EPN and 5-HT in M1, whereas it decreased both DA in dLGN and the DOPAC/DA ratio in the NAc. These changes are difficult to explain because attributing a specific functional meaning to a change in *ex vivo* monoamine tissue levels is very challenging (De Deurwaerdère et al., 2020). Moreover, they show only one time-point of the changes induced by WIN 55,212-2 on both extracellular and intracellular neurotransmitter compartments, different from the microdialysis that measures extracellular time-dependent concentrations (Di Giovanni and Di Matteo, 2013).

Interestingly, we found that WIN 55,212-2 also reduced locomotor activity in a strain-dependent fashion, in agreement with previous observations in Wistar and Fischer 344 rats (Brand et al., 2012). The reduction of both horizontal and vertical locomotor activity in WIN 55,212-2-treated GAERS in hole board is in agreement with previous studies in other general anxiety tests such as the OFT (Järbe et al., 2006) and EPM (Pandolfo et al., 2007). The cannabinoid hypolocomotor effect is mediated by CB1Rs on both GABAergic and glutamatergic inputs to the basal ganglia (De Giacomo et al., 2020) that express high levels of CB1Rs (Mackie, 2005). The locomotor depressant effects of cannabinoids also seem to involve monoamine modulation in the cortico-basal ganglia-thalamocortical loop (Ameri, 1999; Antonazzo et al., 2019). Here, in GAERS, we have shown that WIN 55,212-2 reduced the DOPAC/DA ratio in the NAc and strongly enhanced the 5-HT tissue content in the SN and NA in the EPN. The reduction of the DOPAC/DA ratio was not expected because WIN 55,212-2 has been consistently reported to enhance DA neuronal activity and DA release in the NAc, although preferentially in the shell (Tanda et al., 1997; Polissidis et al., 2013). The reduction in DOPAC/DA ratio in GAERS is perhaps connected to the strong increase in 5-HT tissue content in the SN that is known to mediate DA cell inhibition (Di Giovanni et al., 2008; De Deurwaerdère and Di Giovanni, 2017). The increase in 5-HT and NA levels driven by WIN 55,212-2 might depend on the potentiation of the excitatory effect of CB1Rs of the 5-HT dorsal raphe nucleus (Bambico et al., 2007) and NA locus coeruleus neurons (Gobbi et al., 2005), respectively, compared to NEC rats. The increase of 5-HT in the SN and NA in the EPN may lead to SN reticulata (SNr) GABAergic and EPN neurons inhibition (Di Matteo et al., 2008). Therefore, it can be hypothesized that the WIN 55,212-2 decrease movement depends on reducing the basal ganglia output nuclei, *via* a direct CB1R activation on striatal inhibitory terminals in the SNr (Sales-Carbonell et al., 2013), and an indirect CB1R-mediated increase of NA and 5-HT in the EPN and SN, respectively, in GAERS. The strong sedative effect and the blunt emotional response observed in GAERS compared to NEC may depend respectively on the upregulation/over-function of CB1Rs in motor areas and downregulation of the cannabinoid signals in mood-related areas such as the hippocampus. Considering that WIN 55,212-2 is a CB1/2Rs (Howlett et al., 2002) and functional CB2Rs are expressed not only on microglia (Kelly et al., 2020), but also on neurons of the basal ganglia nuclei (Sánchez-Zavaleta et al., 2018; Zhang et al., 2021), hippocampus (Li and Kim, 2016) and cortex (Morgan et al., 2009), we cannot exclude that the effects observed in this study may also depend on the less investigated CB2Rs.

## Conclusion

In conclusion, the higher level of anxiety observed here in vehicle-treated NEC compared to GAERS rats may be due to a maladaptive response to stress, a critical issue that deserves further investigation. Moreover, we revealed strain-dependent responsiveness to exogenous cannabinoid receptor activation consisting of changes in anxiety-like and motor behaviors, as well as in monoamine levels in a specific mood and motor brain areas, consistent with a sedative and anxiolytic effect in GAERS and NEC rats, respectively.

Furthermore, we show the importance of performing multivariate analysis of behavior *via* T-pattern to avoid misleading interpretation of the disjointed single behaviors by classical quantitative analysis. This type of analysis allowed us to discern the sedative effect of WIN 55,212-2 over its anxiety control in GAERS rats.

Finally, our data add important information to our current understanding of the mechanisms underlying anxiety-related traits in the GAERS model of CAE and their NEC control rats and the ECS abnormalities that are present in these two strains.

## Data Availability Statement

The raw data supporting the conclusions of this article will be made available by the authors, without undue reservation.

## Ethics Statement

The animal study was reviewed and approved by the University Research and Ethics Committee (UREC) and Faculty Research Ethics Committee (FREC) of the University of Malta.

## Author Contributions

GD conceived the study. PD, MC, and GD designed the methodology, performed data analysis, and wrote the manuscript. MR, DC, EP, AC, and PD conducted laboratory-based research and performed data analysis. MC, PD, and AC prepared tables and figures. GD, PD, MC, AC, GC, and VC reviewed and edited the manuscript. All authors have agreed to this manuscript submission for publication.

## Conflict of Interest

The authors declare that the research was conducted in the absence of any commercial or financial relationships that could be construed as a potential conflict of interest.

## Publisher’s Note

All claims expressed in this article are solely those of the authors and do not necessarily represent those of their affiliated organizations, or those of the publisher, the editors and the reviewers. Any product that may be evaluated in this article, or claim that may be made by its manufacturer, is not guaranteed or endorsed by the publisher.

## Abbreviations

AD: anterodorsal nucleus of the thalamus
DA: dopamine
dHP: dorsal part of the hippocampus
DOPAC: 3,4-dihydroxyphenylacetic acid
EPN: entopeduncular nucleus
HPLC: high-pressure liquid chromatography
HVA: homovanillic acid
dLGN: dorsal lateral geniculate nucleus
lSTR and mSTR: lateral and medial striatum
M1: motor cortex
NA: noradrenaline
NAc: nucleus accumbens
NRT: nucleus reticularis thalami
Po: posterior thalamic nucleus
5-HIAA: 5-hydroxy indole-3-acetic acid
5-HT: 5-hydroxytryptamine, serotonin
S1: somatosensorial cortex
SN: substantia nigra
SNc: substantia nigra par compacta
SNr: substantia nigra pars reticulata
VB: ventrobasal complex of the thalamus
V1: the visual cortex.
