# Supplementary material for: Cannabinoid 1/2 Receptor Activation Induces Strain-Dependent Behavioral and Neurochemical Changes in Genetic Absence Epilepsy Rats From Strasbourg and Non-epileptic Control Rats
Source: Front Cell Neurosci. 2022 May 23;16:886033. doi: 10.3389/fncel.2022.886033 (PMC9169225; doi:10.3389/fncel.2022.886033)
Supplement: Supplementary file 1 [file Data_Sheet_1.docx]

**
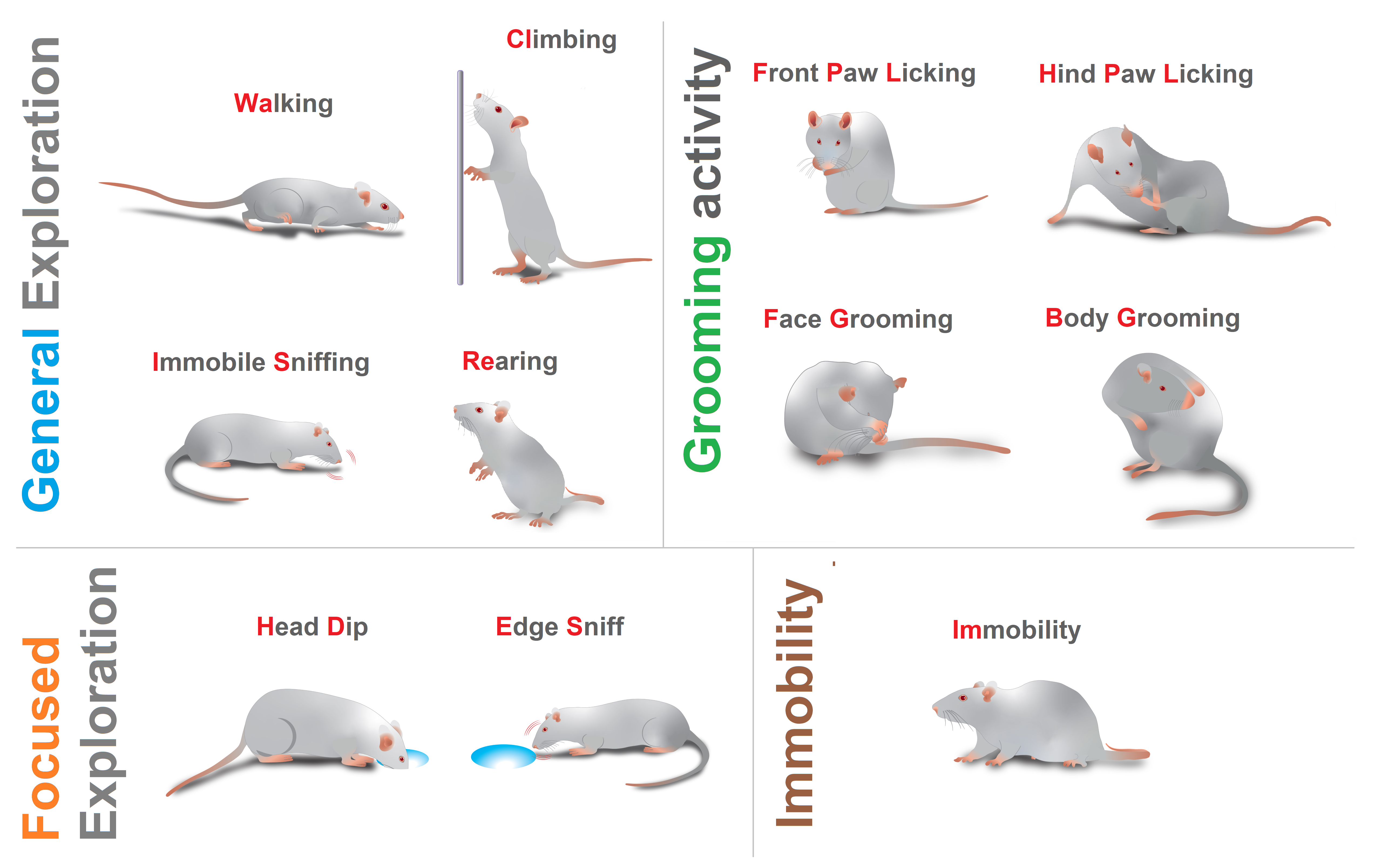
**

**Supplementary Figure 1**. **Ethogram of GAERS and NEC rat behavior in the hole-board apparatus.**

**General exploration**: Walking (**Wa**) = the rat walks around sniffing the environment; Climbing (**Cl**) = the rat maintains an erect posture leaning against the Plexiglas wall; Immobile-Sniffing (**IS**): the rat sniffs the environment standing on the ground; Rearing (**Re**) = rat maintains an erect posture without leaning against the Plexiglas box. **Focused exploration**: Head-Dip (**HD**) = rat puts its head into one of the four holes; Edge-Sniff (**ES**): rat sniffs the border of one of the four holes. **Grooming activity**: Front Paw Licking (**FPL**): rat licks or grooms its forepaws; Hind Paw Licking (**HPL**): rat licks or grooms its hind paws; Face Grooming (**FG**): rat rubs its face (ears, mouth, vibrissae, eyes); Body-Grooming (**BG**): rat licks its body combing its fur with fast movements of incisors. **Immobility** (**Im**): rat maintains a fixed posture and no movements are observed.

**
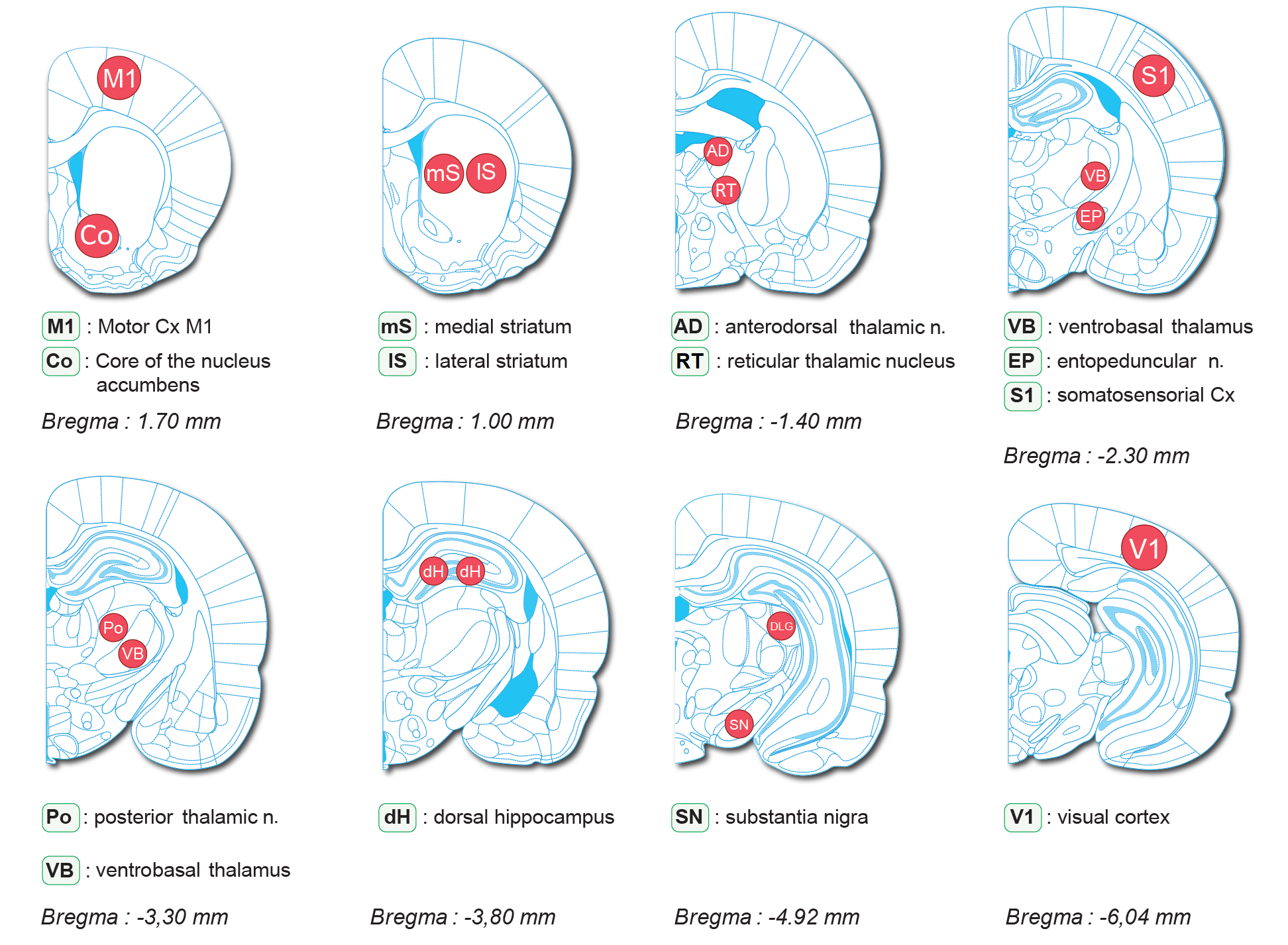
Supplementary Figure 2. Location of the punches used in different brain regions to detect monoamines levels in WIN 55,212-2- and vehicle-treatyed of GAERS and NEC rats.** GAERS and NEC rats’ brains were collected after 30 min the administration of WIN 55,212-2 (WIN; 2 mg/kg, i.p.) and its vehicle and stored in a freezer at -80°C until analyzed. Tissue samples were taken from the left and right cerebral hemispheres. **AD**, anterodorsal thalamic nucleus; **dH** (dorsal part of the hippocampus); **EPN**, entodepuncular nucleus; **dLGN**, dorsal lateral geniculate nucleus; **lS** and **mS** lateral and medial striatum; **M1**, motor cortex; **Co,** core of the nucleus accumbens; **RT**, reticular thalamic nucleus; **Po**, posterior thalamic nucleus; **S1**, somatosensorial cortex, **SN**, substantia nigra; **VB**, ventrobasal complex of the thalamus; **V1**, visual cortex.
